# Regulation of Phosphatidylinositol Synthesis in Human Primordial Placenta

**DOI:** 10.3390/biom16020300

**Published:** 2026-02-14

**Authors:** Bence Kovács, Zoltán Erdélyi, Gergely Asbóth, Gábor Gimes, Balázs Mészáros, Zsófia Erdélyi, Tamás Marton, Nándor Ács, Dorina Supák, Sándor Valent, Zoltán Kukor

**Affiliations:** 1 Department of Obstetrics and Gynecology, Semmelweis University, Üllői út 78/a, 1082 Budapest, Hungary; kovacs.bence@semmelweis.hu (B.K.); erdelyi.zsofia@stud.semmelweis.hu (Z.E.);; 2 Department of Molecular Biology, Institute of Biochemistry and Molecular Biology, Semmelweis University, Tűzoltó u. 37-47, 1093 Budapest, Hungarykukor.zoltan@semmelweis.hu (Z.K.); 3 Institute of Pharmacogenetics, University Hospital Essen, 1085 Essen, Germany; 4 Department of Pathology, Forensic and Insurance Medicine, Semmelweis University, 1093 Budapest, Hungary

**Keywords:** human primordial placenta, phosphatidylinositol synthesis, G protein, signal transduction

## Abstract

Phosphatidylinositol and its derivatives are essential components of cell membranes and play pivotal roles in growth signaling pathways. In the human primordial placenta, phosphatidylinositol synthesis is catalyzed by phosphatidylinositol synthase (PIS) and the phosphatidylinositol-exchange enzyme (IE), both of which require divalent cations. We investigated whether GTP-binding proteins modulate this biosynthetic process. Incorporation of [^3^H]inositol into phosphatidylinositol was measured in trophoblast tissue and microsomes from 8 to 10-week placentas. Our results demonstrate that Mn^2+^ strongly enhances phosphatidylinositol synthesis, and stimulation with AlF_4_^−^ further increases incorporation rates by up to 2.5-fold. In contrast, Mg^2+^ combined with the non-hydrolyzable GTP analog GIDP elevated synthesis by 58%, whereas Mn^2+^ plus GIDP reduced incorporation by 30%. Complementary in silico protein–protein interaction analyses suggest that G-proteins may directly associate with inositol-exchange enzymes, providing a potential mechanism for the observed regulatory effects. These findings indicate that phosphatidylinositol synthesis is modulated in a manner consistent with G-protein involvement, with distinct effects depending on the prevailing enzymatic pathway. We propose that rapid trophoblast proliferation may involve feedback mechanisms mediated by distinct G-protein subtypes acting on early steps of the phosphatidylinositol cycle.

## 1. Introduction

Phosphatidylinositol (PI) serves as the precursor for phosphatidylinositol phosphates (PIPs), a family of low-abundance but highly regulatory lipids that control membrane trafficking, signaling, and cytoskeletal organization. Tight regulation of PI synthesis is therefore critical for maintaining phosphoinositide homeostasis, particularly in highly dynamic tissues such as the placenta [1]. During placental development, cell division and migration are highly intensive processes, especially in the period following implantation [2,3,4,5,6]. The organ consists of two major components: trophoblast cells and the extraembryonic mesoderm. Extravillous trophoblast cells possess remarkable migratory and invasive properties that are essential for early development and for interactions with maternal tissues [7,8,9]. The rapid growth of the placenta is supported by active growth signaling pathways that regulate cellular dynamics [10,11].

Central to these growth signaling pathways are G-proteins, among which Ras proteins [12,13] play a central role in regulating cell growth and differentiation, while the Gαs and Gαq subunits control cAMP- and Ca^2+^-dependent signaling through the activation of adenylate cyclase and phospholipase C, respectively [14,15,16]. These proteins are crucial regulators of cell proliferation and migration and thus represent fundamental determinants of placental development.

To investigate G-protein function, methods that maintain these proteins in a continuously active state are widely used. Aluminum tetrafluoride [AlF_4_^−^] is capable of stabilizing the Gα subunit in its active conformation, whereas non-hydrolyzable GTP analogs—such as GTPγS or GIDP—prevent GTP hydrolysis and thereby induce sustained activation [17].

Research focusing on the investigation of growth signaling pathways has become a major focus in recent decades. Within these pathways, the interconversion of phosphatidylinositol phosphates plays a central role in signal transduction; however, the very first step—the incorporation of inositol into lipids, i.e., the synthesis of phosphatidylinositols—remains poorly characterized, and its regulatory mechanisms are largely unknown [18]. This gap in knowledge is particularly noteworthy given that phosphatidylinositol synthesis represents the fundamental starting point for the formation of downstream phosphatidylinositol phosphates, which play key roles in numerous cellular functions.

In our previous work, we demonstrated that in the presence of Mg^2+^, both GTP and GIDP significantly increase phosphatidylinositol synthesis, suggesting that the interactions between G-protein and divalent cations may represent a key factor in the regulation of placental phosphatidylinositol metabolism [19]. This observation provides the rationale for the present study, in which we examine G-protein activity in the presence of Mn^2+^, with particular emphasis on the regulation of PIS and IE activities [16,20].

Human trophoblasts synthesize phosphatidylinositol via two distinct pathways. In pathway one, phosphatidylinositol synthase (PIS) catalyzes the reaction CDP-diacylglycerol + inositol → phosphatidylinositol + CMP [21]. The second route is recently identified inositol exchange enzyme (IE) in human trophoblasts [19], which catalyzes the phosphatidyl-X + inositol → PI + X reaction, where “X” can represent various substrates such as choline or ethanolamine. PIS activity increases with Mg^2+^ concentrations up to 100 mM in vitro, a range that exceeds physiological free Mg^2+^ levels but has been widely used to define the maximal catalytic capacity and cation dependence of the enzyme. Mn^2+^ enhances PIS activity up to 1 mM, but has an inhibitory effect at higher concentrations. In contrast, IE activity is inhibited by high Mg^2+^ levels, while increasing Mn^2+^ concentrations will stimulate its activity. Consequently, based on their established divalent cation dependencies, conditions of high Mn^2+^ are expected to favor inositol-exchange-mediated incorporation, whereas high Mg^2+^ conditions preferentially support phosphatidylinositol synthase activity [19].

The aim of our study is to elucidate the regulation of this early synthetic step using a human trophoblast model. We paid particular attention to understanding the role of small GTP-binding proteins in the enzymatic processes and exploring how phosphatidylinositol synthesis may be integrated into feedback regulatory mechanisms. The insight gained from this work may not only enhance our understanding of placental development but may also provide new perspectives for investigating the regulation of tumorigenic processes, potentially identifying novel therapeutic targets [11].

## 2. Methods

### 2.1. Materials

[3H]inositol [50 µCi/mmol; 1.85 GBq/mmol] was obtained from ICN [Costa Mesa, CA, USA]. Tris base [Tris[hydroxymethyl]aminomethane], HCl, KCl, Na_2_HPO_4_, KH_2_PO_4_, NaHCO_3_, MgSO_4_, CaCl_2_, chloroform, methanol, acetic acid, Triton X100, toluene, glucose, and sucrose were purchased from REANAL [Budapest, Hungary]. Dithiothreitol (DTT) and NaCl were from Sigma Chemical Co. (Budapest, Hungary). Phenylmethylsulphonyl fluoride (PMSF) and HEPES were from Calbiochem (La Jolla, CA, USA). The concentrations of NaF (5 mM) and AlCl_3_ (100 µM) used to generate AlF_4_^−^ were selected based on widely used biochemical protocols for G-protein activation and were applied consistently across all experimental conditions.

### 2.2. Placental Tissue Collection

Primordial human placental tissues were obtained from legally performed social terminations of pregnancy (8–10 weeks of gestation) following surgical evacuation at the Department of Obstetrics and Gynecology, Semmelweis University (Budapest, Hungary). The use of tissues for research purposes was approved by the Ethics Committee of the Medical Section of the Hungarian Academy of Sciences (approval number: BM/15435-1/2023; approval date: 27 June 2023). Informed consent was obtained from all women who participated in the study prior to sample collection. None of the patients received myo-inositol or D-chiro-inositol therapy. Exclusion criteria were smoking, polycystic ovary syndrome (PCOS), diabetes mellitus, hypertension, known genetic disorders in the family (in first-degree relatives), and previous pregnancy complications (e.g., gestational diabetes mellitus, preeclampsia, or fetal genetic abnormalities). For each experimental series, trophoblast tissue obtained from three independent pregnancy terminations (8–10 weeks of gestation) was pooled prior to incubation or microsome preparation to ensure sufficient material and to reduce variability arising from tissue processing. As a consequence, individual donors were not treated as independent biological replicates, and inter-individual variability was not assessed in this study.

### 2.3. Microsome Preparation

Trophoblast tissue was homogenized in two volumes of ice-cold homogenizing solution containing 300 mM sucrose, 0.5 mM DTT, 5 mM EDTA, and 50 mM Tris/HCl (pH 7.4). The homogenate was filtered through nylon mesh, and heavy particulate material and mitochondria were sedimented at 15,000× *g* for 30 min. The supernatant was then centrifuged at 100,000× *g* for 60 min to obtain the microsomal pellet, which was resuspended in homogenizing solution. Both tissue and microsomes were incubated in Hank’s solution at 37 °C under shaking conditions.

Tissue activity was expressed as counts per minute (cpm) of [^3^H]-inositol/600 mg tissue, whereas microsomal activity was expressed as cpm [^3^H]-inositol/mg protein × minute.

### 2.4. Protein Determination

Protein concentration was measured using the conventional method of Lowry et al., with bovine serum albumin serving as the standard [22]. Relative activities were calculated in comparison to the control. The separation and purity of phosphatidylinositol and inositol were verified by thin-layer chromatography using a phosphatidylinositol standard [23].

### 2.5. Protein–Protein Interactions

Protein biosynthesis inhibitors have long been used to probe cellular regulatory mechanisms and protein interaction dynamics [24]. Protein–protein interaction probabilities were computed using SENSE-PPI, D-Script, Topsy-Turvy, and ProteinPrompt. SENSE-PPI and D-Script generate interaction scores from deep-learning-derived sequence embeddings, with D-Script additionally inferring predicted residue–residue contact maps to incorporate structural constraints. ProteinPrompt directly predicts interaction probabilities from primary amino acid sequences without explicit structural modeling. Topsy-Turvy applies a contrastive learning framework that integrates co-evolutionary and structural compatibility features to estimate interaction likelihoods. ProteinPrompt predictions were obtained using the publicly available web server (https://proteinformatics.uni-leipzig.de/protein_prompt/; accessed on 2 December 2025). D-Script and Topsy-Turvy predictions were generated via the authors’ web interface (https://dscript.csail.mit.edu; accessed on 2 December 2025), and SENSE-PPI predictions were computed using the original pretrained model provided by the authors (https://pypi.org/project/senseppi/; accessed on 2 December 2025).

### 2.6. Data Analysis

The results were statistically evaluated by the GraphPAD InStat 1.2 program. The difference was considered to be significant if the “*p*” value was <0.05 (Bonferroni multiple comparison). The graphic presentations were made with the help of Microsoft Office PowerPoint 2016 and Microsoft Office Excel 2016 programs. Data are presented as mean ± standard deviation (SD). Unless otherwise stated, statistical comparisons were performed using two-tailed Student’s *t*-tests for pairwise comparisons against control conditions. Prior to parametric testing, data distributions were assessed for approximate normality and homogeneity of variance; no gross deviations were observed. Each comparison represented a predefined, hypothesis-driven contrast, and therefore no formal correction for multiple comparisons was applied. Statistical significance was defined as *p* < 0.05. Reported *n* values correspond to independent technical replicate incubations performed on pooled trophoblast material derived from three independent donors and do not represent biological replication at the donor level.

## 3. Results

### 3.1. Effect of Divalent Cations and AlF_4_^−^ on Phosphatidylinositol Synthesis

Trophoblast fragments [400 mg] were incubated with [3H]inositol for 60 min at 37 °C. Phosphatidylinositol labeling was not significantly altered (*p* > 0.05) by 5.0 mM NaF or 100 µM AlCl_3_ alone under Ca^2+^ or Mg^2+^ conditions. In contrast, the combined application of NaF and AlCl_3_ (AlF_4_^−^) resulted in a marked stimulation of phosphatidylinositol labeling in the presence of Mn^2+^ (Table 1). In the presence of 1 mM CaCl_2_, these agents likewise did not change phosphatidylinositol radioactivity compared with controls. By contrast, 1 mM MnCl_2_ markedly increased phosphatidylinositol labeling, more than 8-fold (*p* < 0.001). Addition of AlF_4_^−^ further enhanced Mn^2+^-dependent activity by over 2.5-fold (*p* < 0.01) (Table 1). Importantly, neither fluoride nor aluminum alone reproduced the effect of AlF_4_^−^, indicating that the observed stimulation requires formation of the AlF_4_^−^ complex rather than independent actions of its components. In this experimental series, each condition represents four independent measurements (*n* = 4), each performed on homogenates prepared from three different primordial placentas, resulting in a total of 12 individual placentas analyzed per condition.

### 3.2. AlF_4_^−^ Increases Trophoblast Phosphatidylinositol Synthesis with Mn^2+^, but Not with Mg^2+^

**Figure 1:**
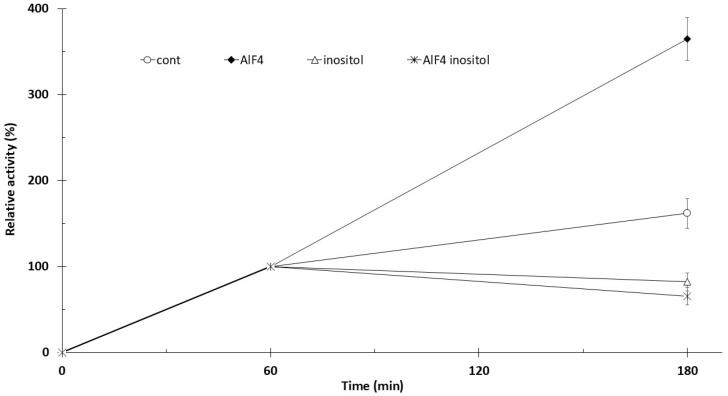
Effect of AlF_4_^−^ on trophoblast phosphatidylinositol synthesis in 1 mM Mn^2+^. Trophoblasts were preincubated for 60 min at 37 °C with 1 mM MnCl_2_ and 5 µCi [3H]inositol for 60 min; the following agents were added: AlF4 (100 µM AlCl_3_ + 5 mM NaF). Control samples received no additions. *n* = 4 ± SD.

Trophoblast samples were preincubated with 5 µCi [^3^H]inositol for 60 min at 37 °C in the presence of 1 mM MnCl_2_, followed by an additional 120 min incubation with the agents indicated in Figure 1. The absolute phosphatidyl-[^3^H]inositol-associated radioactivity of control samples was 39,218 cpm/600 mg tissue (60 min, 37 °C), and all data shown in Figure 1 are expressed relative to this control value (100%). For this measurement, tissues from 12 different primordial placentas (4 × 3) were used. Phosphatidyl-[^3^H]inositol-associated radioactivity measured at 60 min increased by approximately 60% after 180 min of incubation. The addition of 100 µM AlF_4_^−^ increased radioactivity at 180 min by approximately 2.3-fold compared with the 180-min control. Administration of 10 mM inositol at 60 min reduced the 60-min labeling measured at 180 min by 18% (*p* < 0.01), whereas combined treatment with inositol and AlF_4_^−^ resulted in a 35% reduction (*p* < 0.01).

**Figure 2:**
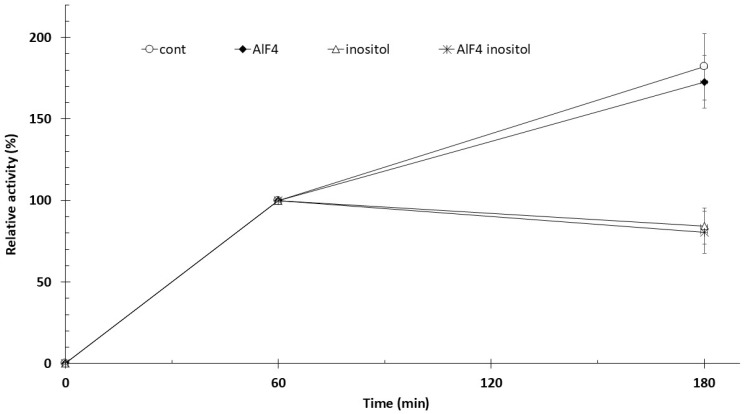
Effect of AlF_4_^−^ on trophoblast phosphatidylinositol synthesis in 1 mM Mg^2+^. Trophoblasts were preincubated for 60 min at 37 °C with 1 mM MgCl_2_ and 5 µCi [3H]inositol for 60 min; the following agents were added: AlF4 (100 µM AlCl_3_ + 5 mM NaF). Control samples received no additions. *n* = 4 ± SD.

Trophoblast samples were preincubated with 5 µCi [^3^H]inositol for 60 min at 37 °C in the presence of 1 mM MgCl_2_, followed by an additional 120 min incubation with the agents indicated in Figure 2. The absolute mean radioactivity of the control samples (60 min incubation) was 14,717 cpm [3H]inositol/600 mg tissue. 12 different primordial placentas were used for this experiment. Phosphatidyl-[^3^H]inositol-associated radioactivity measured at 60 min increased by approximately 80% after 180 min of incubation. The addition of 100 µM AlF_4_^−^ enhanced radioactivity at 180 min by approximately 1.74-fold compared with the control. AlF_4_^−^ did not significantly alter the rate of phosphatidylinositol synthesis. Administration of 10 mM inositol at 60 min reduced the 60-min labeling measured at 180 min by 16% (*p* < 0.01), whereas combined treatment with inositol and AlF_4_^−^ resulted in a 19% reduction (*p* < 0.01).

### 3.3. AlF_4_^−^ Increases Microsomal Phosphatidylinositol Synthesis with Mn^2+^

**Figure 3:**
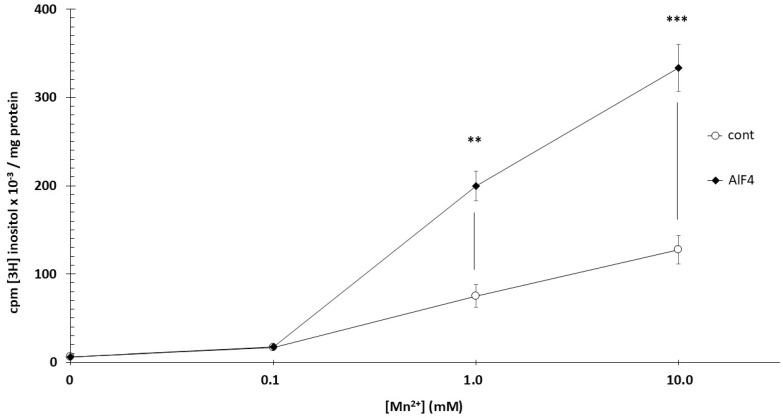
Incorporation of [3H]inositol into phosphatidylinositol in trophoblast microsomes. 60 min incubation, 37 °C, pH = 7.4. AlF4: 100 µM AlCl_3_ + 5 mM NaF; *n* = 4 ± SD. ** *p* < 0.01; *** *p* < 0.001.

Microsomes were incubated for 60 min at 37 °C with [3H]inositol. Both phosphatidylinositol synthase and the inositol-exchange enzyme reach maximal activity at millimolar Mn^2+^ concentrations. At 0.1 mM Mn^2+^, activity was already significantly elevated compared with baseline (*p* < 0.01), with further increases observed at 1 and 10 mM Mn^2+^. At 10 mM Mn^2+^, activity exceeded control by more than 20-fold, reflecting a non-physiological but experimentally useful condition that strongly biases phosphatidylinositol incorporation toward Mn^2+^-dependent enzymatic routes. AlF_4_^−^ (100 µM) enhanced phosphatidylinositol synthesis only at millimolar Mn^2+^ concentrations: at 1 mM Mn^2+^, activity increased 2.62-fold (*p* < 0.01), and at 10 mM Mn^2+^, 2.55-fold (*p* < 0.001) (Figure 3). In this experiment, tissues from a total of 12 different primordial placentas were used.

### 3.4. In the Presence of Mn^2+^, GTP Reduces Phosphatidylinositol Synthesis

**Figure 4:**
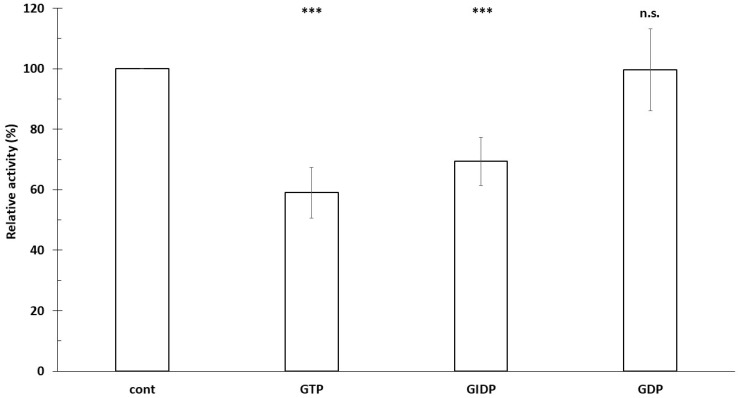
Effect of GTP, GIDP, and GTP on the incorporation of [3H]inositol into phosphatidylinositol in trophoblast microsomes. 60 min incubation, 37 °C, pH = 7.4, 10 mM Mn^2+^, AlF4: 100 µM AlCl_3_ + 5 mM NaF; *n* = 4 ± SD. *** *p* < 0.001, n.s.: not significant compared to control.

The absolute phosphatidyl-[^3^H]inositol-associated radioactivity of control samples was 94,528 cpm/mg protein, and all data shown in Figure 4 are expressed relative to this control value (100%). For this measurement, tissues from 12 different primordial placentas (4 × 3) were used. PIS microsomal activity is significantly increased by GIDP (57%), but not significantly by GTP (33%) [19]. We therefore examined the effects of GTP, GIDP, and GDP on [^3^H]inositol incorporation into phosphatidylinositol in the presence of 10 mM Mn^2+^ (IE activity). Both GTP (39.9%, *p* < 0.001) and GIDP (30.6%, *p* < 0.001) significantly decreased [^3^H]inositol incorporation, whereas no significant change was observed with GDP (Figure 4).

### 3.5. Protein–Protein Interactions

As a next step, to explore the protein–protein interactions behind the above-described effects, we performed an in silico interaction screen using the SENSE-PPI^1^, D-Script^2^, Topsy-Turvy^3^, and ProteinPrompt^4^ models. The sequence-based approaches SENSE-PPI and D-Script did not identify small GTPases or heterotrimeric G-protein α subunits as interaction candidates with enzymes involved in phosphatidylinositol metabolism. In contrast, the contrastive-learning-based models Topsy-Turvy and ProteinPrompt yielded higher predicted interaction probabilities for small GTPases and phosphatidylinositol synthase (CDP-diacylglycerol–inositol 3-phosphatidyltransferase); however, none of these predictions exceeded the predefined confidence cutoff of 0.8 for high-confidence predictions (Table 2). The resulting predictions are consistently hypothesis-generating, suggesting that G-proteins directly influence phosphatidylinositol homeostasis, providing a rationale for further experimental investigation. Although these predictions require experimental validation, they are consistent with our biochemical data, suggesting G-protein-dependent regulation of phosphatidylinositol synthesis.

## 4. Discussion

Our findings demonstrate that phosphatidylinositol synthesis in the human primordial placenta is subject to multilayered regulation by G-protein signaling and that the control arises from differential modulation of the two enzymatic components of the phosphatidylinositol cycle, namely phosphatidylinositol synthase and the inositol-exchange (scramblase-like) machinery [19]. The strong stimulation of phosphatidylinositol synthesis by Mn^2+^ is consistent with previous observations and reflects the preferential activation of the inositol-exchange pathway under these conditions [21]. The stimulatory effect of AlF_4_^−^ on PI synthesis in the presence of Mn^2+^ may reflect the stabilization of a GTPase-like transition state in a regulatory protein, coupled with Mn^2+^-dependent facilitation of catalytically competent conformations of the PI-synthesizing machinery. This interpretation remains speculative and is presented here as a working hypothesis.

Under Mg^2+^-dominant conditions, which favor phosphatidylinositol synthase activity, the non-hydrolyzable GTP analog GIDP increased phosphatidylinositol synthesis, whereas engagement of the inositol-exchange pathway in the presence of Mn^2+^ resulted in reduced incorporation. In contrast, AlF_4_^−^ selectively enhanced phosphatidylinositol synthesis when inositol exchange was active, while exerting no detectable effect on phosphatidylinositol synthase-dominated flux. This bidirectional regulation indicates that G-protein activation can exert opposing effects on distinct modules of the phosphatidylinositol cycle, depending on the prevailing enzymatic pathway [18,25].

To explore a potential mechanistic basis for these observations, we performed in silico protein–protein interaction analyses focusing on inositol-exchange enzymes, including members of the phospholipid scramblase family [26]. While sequence-based prediction models did not identify G-proteins as prominent interaction partners, structure-informed approaches suggested that specific Gα subunits may directly associate with phospholipid scramblases. Notably, structure-informed predictions suggested potential associations between PLSCR1 and selected Gα subunits, particularly members of the Gαi family, although these interactions remain hypothetical, while additional moderate and still hypothetical predictions involved Gαq and Gα13. Although these predictions require experimental validation, they provide a plausible framework for interpreting the opposing effects of G-protein activators observed in our biochemical assays [26,27].

One possible interpretation is that stabilization of certain Gα subunits by GIDP could be associated with inhibition of the inositol-exchange step; however, direct causal links cannot be established based on the present data. Conversely, AlF_4_^−^ activates a broader spectrum of G-proteins, including Gαq-family members, which are linked to Ca^2+^ signaling. Given the Ca^2+^ dependence of phospholipid scramblase activity, Gαq-mediated signaling offers a potential mechanism for the pronounced stimulation of inositol exchange by AlF_4_^−^. However, the current data do not allow definitive assignment of specific Gα isoforms as direct regulators, and additional routes of regulation cannot be excluded. Indeed, in silico predictions also suggested potential interactions between Gα subunits and PIK3IP1, indicating that G-proteins may influence phosphatidylinositol metabolism through multiple, parallel mechanisms.

Reinterpretation of our earlier observations in light of this model suggests that the AlF_4_^−^-induced increase in phosphatidylinositol synthesis reflects enhanced inositol exchange rather than generalized activation of phosphatidylinositol synthase. Likewise, the strong Mn^2+^ dependence of microsomal labeling is consistent with a model in which inositol-exchange-mediated incorporation contributes substantially under these conditions, as suggested by prior biochemical characterization [19,28].

From a broader perspective, the placenta is characterized by rapid membrane expansion and high phosphoinositide turnover, and G-protein-dependent modulation of phosphatidylinositol availability may represent a mechanism for coupling lipid metabolism to proliferative and migratory signaling [29,30,31]. Given that Gαi- and Gαq-mediated pathways are frequently dysregulated in cancer, a similar dual-mode regulatory architecture may operate in other rapidly dividing cells, suggesting a hypothesis that regulation of the inositol-exchange step by G-proteins could contribute to proliferative signaling under pathological conditions. Experimental validation will be required to test this possibility [32]. If validated, regulation of the inositol-exchange step by G-proteins could represent a previously unrecognized point of control within the phosphatidylinositol cycle, with implications for both physiological development and pathological growth states. At present, these data do not allow definitive assignment of individual Gα isoforms as direct regulators [33,34].

### Strengths and Limitations

This study benefits from the use of human primordial placental trophoblast tissue, providing a physiologically relevant model for investigating early trophoblast signaling. Quantitative assessment of phosphatidylinositol synthesis was achieved through direct [^3^H]inositol incorporation, enabling reliable measurement of enzymatic activity. The controlled activation of G-proteins by AlF_4_^−^ allowed mechanistic insights into signaling regulation, while parallel experiments in intact trophoblast tissue and microsomal fractions revealed compartment-specific differences in phosphatidylinositol synthesis.

A number of limitations should be acknowledged. Firstly, samples from multiple pregnancy terminations were pooled; inter-individual variability cannot be completely excluded. Secondly, the in vitro incubation conditions may not fully reflect the complexity of the in vivo placental environment. Additionally, phosphatidylinositol synthesis was quantified using [^3^H]inositol incorporation, which does not discriminate between phosphatidylinositol synthase-mediated synthesis and inositol-exchange-mediated incorporation. Therefore, conclusions regarding the relative contribution of these pathways are inferred from established cation dependencies and prior substrate-specific studies, rather than directly demonstrated in the present experiments. Orthogonal approaches such as substrate-defined assays, selective inhibition or depletion, or lipidomic tracing will be required to experimentally resolve pathway-specific flux. Importantly, the use of AlF_4_^−^ and non-hydrolyzable GTP analogs does not allow discrimination between individual GTPase families or Gα isoforms. Therefore, the proposed involvement of specific G-proteins should be regarded as hypothesis-generating and requires targeted experimental validation. Whilst the effects of Mn^2+^ and AlF_4_^−^ were clearly demonstrated, the precise molecular targets and downstream pathways remain to be identified.

Building on these limitations, future studies should focus on experimentally validating the proposed interactions between Gα subunits and phospholipid scramblases, clarifying the potentially opposing roles of Gαi and Gαq in regulating PLSCR activity, and determining the contribution of Ca^2+^ binding to AlF_4_^−^-mediated effects. Extending these analyses to other high-turnover tissues and integrating lipidomic approaches with spatially resolved measurements of phosphatidylinositol turnover will be essential to define tissue specificity and compartmental regulation. In summary, these findings are hypothesis-generating and support a model in which G-protein signaling exerts isoform-specific and directional control over distinct modules of the phosphatidylinositol cycle, with broader implications for membrane dynamics and proliferative signaling.

## 5. Conclusions

Our study demonstrates that phosphatidylinositol synthesis in the human primordial placenta is functionally modulated by signaling mechanisms consistent with G-protein activation, in a divalent cation-dependent manner. The data also indicate that G-protein activation exerts opposing effects on distinct modules of the phosphatidylinositol cycle, with phosphatidylinositol synthase and the inositol-exchange pathway responding differentially to GTP analogs and AlF_4_^−^. The predicted protein–protein interactions between Gα subunits and inositol-exchange enzymes were identified using multiple computational models. While these predictions provide a rationale for potential regulatory interactions, we emphasize that they remain hypothetical and require experimental validation through direct biochemical or biophysical assays in future studies. These observations reveal an additional regulatory layer in placental lipid metabolism and suggest that G-protein-dependent control of early phosphatidylinositol synthesis may contribute to the coordination of membrane dynamics with proliferative signaling.

## Figures and Tables

**Table 1:** Labeling of trophoblast phosphatidyl-[3H]inositol. 400 mg trophoblast, 60 min, 37 °C, pH = 7.4. F: 5 mM NaF; Al: 100 µM AlCl_3_; AlF4: 100 µM AlCl_3_, 5 mM NaF; *n* = 4 ± SD.

	cpm Phosphatidyl-[3H]inositol/400 mg Trophoblast ± SD			
	---	1 mM Ca^2+^	1 mM Mn^2+^	1 mM Mg^2+^
control	3065 ± 138	3221 ± 331	25,140 ± 853	8314 ± 942
F	2770 ± 132	2612 ± 144	28,931 ± 4717	8531 ± 584
Al	2718 ± 195	2585 ± 156	26,533 ± 3835	8166 ± 907
AlF4	2721 ± 142	2588 ± 111	66,079 ± 7550	8325 ± 974

**Table 2:** Potential protein–protein interactions.

Protein 1	Protein 2	Interaction Score	
ANO6 (910aa)	All G-proteins	<0.5	D-SCRIPT
	All G-proteins	<0.5	SENSE-PPI
	GNAI2_318	0.749	Topsy-Turvy
	GNAI1	0.6400	ProteinPrompt
PLSCR1 (318aa)	All G-proteins	<0.5	D-SCRIPT
	All G-proteins	<0.5	SENSE-PPI
	GNAI2_318	0.743	Topsy-Turvy
	GNAI2	0.9800	ProteinPrompt
	GNAI1	0.9040	ProteinPrompt
	GNA13	0.8893	ProteinPrompt
	GNAQ	0.8840	ProteinPrompt
PLSCR2 (297aa)	All G-proteins	<0.5	D-SCRIPT
	All G-proteins	<0.5	SENSE-PPI
	GNAI2_318	0.685	Topsy-Turvy
	GNAI2	0.6467	ProteinPrompt
PLSCR3 (295aa)	All G-proteins	<0.5	D-SCRIPT
	All G-proteins	<0.5	SENSE-PPI
	GNA13_2	0.658	Topsy-Turvy
	GNAI2	0.6080	ProteinPrompt
PLSCR4 (329aa)	All G-proteins	<0.5	D-SCRIPT
	All G-proteins	<0.5	SENSE-PPI
	GNA11	0.680	Topsy-Turvy
	GNAI2	0.6907	ProteinPrompt
PLSCR4 (271aa)	All G-proteins	<0.5	D-SCRIPT
	All G-proteins	<0.5	SENSE-PPI
	GNAI2	0.647	Topsy-Turvy
	GNAI2	0.636	ProteinPrompt

## Data Availability

The original contributions presented in this study are included in the article. Further inquiries can be directed to the corresponding author(s).
